# Targeting DNA Damage Response-Mediated Resistance in Non-Small Cell Lung Cancer: From Mechanistic Insights to Drug Development

**DOI:** 10.3390/curroncol32070367

**Published:** 2025-06-23

**Authors:** Xue Gong, Yongzhao Zhou, Yi Deng

**Affiliations:** Integrated Care Management Center, Institute of Respiratory Health and Multimorbidity, West China Hospital, Sichuan University, Chengdu 610041, China; xuegong@wchscu.cn (X.G.); yongzhaozhou001@wchscu.cn (Y.Z.)

**Keywords:** non-small cell lung cancer, DNA damage response, drug resistance, radiotherapy, chemotherapy, targeted therapy, homologous recombination, non-homologous end joining

## Abstract

Non-small cell lung cancer (NSCLC) remains a major contributor to cancer-related deaths worldwide, with therapeutic resistance presenting a critical clinical hurdle. The DNA damage response (DDR) constitutes a sophisticated cellular framework that detects, signals, and repairs genetic lesions to preserve genomic stability. While the DDR plays a crucial role in determining the efficacy of radiotherapy and chemotherapy, current research primarily focuses on direct DDR inhibitors, often overlooking the broader regulatory networks that modulate DDR activity. This review aims to comprehensively analyze the upstream and downstream pathways governing DDR in NSCLC, highlighting key molecular regulators, signaling interactions, and potential feedback mechanisms contributing to therapy resistance. By identifying novel regulatory targets and clinically relevant biomarkers, we propose innovative therapeutic strategies to enhance treatment efficacy. Our approach seeks to bridge the gap between DDR dysregulation and precision oncology, offering new perspectives on overcoming resistance and improving patient outcomes in NSCLC.

## Simple Summary

Therapeutic resistance to radiotherapy and chemotherapy remains a major challenge in non-small cell lung cancer treatment, limiting the long-term efficacy of conventional therapies. This review provides a comprehensive overview of DNA repair regulation beyond canonical pathways, elucidating novel upstream modulators, downstream effectors, and the intricate network of their interactions. We further highlight potential therapeutic targets, emerging treatment strategies, and current obstacles to clinical translation. These insights offer innovative directions to refine precision medicine approaches and contribute to the development of more effective, individualized therapies for patients facing resistant disease.

## 1. Introduction

Lung cancer remains the leading cause of cancer-related mortality worldwide [1]. In 2022, it accounted for approximately 1.82 million deaths globally, and this number is projected to rise to 3.55 million by 2050 if current trends persist [2]. Non-small cell lung cancer (NSCLC) represents the predominant subtype, comprising nearly 85% of all diagnosed lung malignancies [3]. As classified by the World Health Organization (WHO), NSCLC includes adenocarcinoma, squamous cell carcinoma, and large cell carcinoma [4]. Its incidence is strongly linked to various risk factors, including tobacco use, air pollution, genetic susceptibility, and environmental exposures [4]. The classical therapies for NSCLC include surgery, standard radiotherapy, and chemotherapy [5]. Although significant progress has been made with targeted therapies (e.g., EGFR and ALK inhibitors), immunotherapy (e.g., PD-1/PD-L1 blockade), and combinatorial strategies, the five-year survival rate remains suboptimal [5]. A major barrier to improving patient prognosis is the emergence of resistance mechanisms, including enhanced DNA repair processes, increased drug efflux, activation of alternative signaling pathways, genetic mutations, and immune system evasion [6]. Addressing these resistance mechanisms is crucial for improving treatment outcomes and long-term survival.

The DNA Damage Response (DDR) is an intricate regulatory system that enables cells to recognize and repair genetic damage, thereby preserving genomic integrity [7]. In NSCLC, dysregulation of DDR pathways contributes to genomic instability, accelerating tumor progression. Research indicates that nearly 49.6% of NSCLC patients possess deleterious DDR mutations, which are linked to resistance to chemotherapy, radiotherapy, targeted therapy, and immunotherapy [7]. These mutations not only elevate tumor mutational burden but also correlate with poor prognosis in advanced lung cancer cases [8,9]. Recent breakthroughs, particularly in single-cell RNA sequencing, have provided deeper insight into the intricate relationship between DDR dysfunction and therapy resistance [10]. Findings suggest that aberrant DDR activation enhances the repair of treatment-induced DNA damage while modulating cell cycle checkpoints and apoptotic mechanisms, ultimately enabling tumor cells to evade cytotoxic stress [10]. This has fueled considerable interest in exploiting DDR deficiencies as therapeutic vulnerabilities. A notable example is the clinical application of synthetic lethality, wherein poly (ADP-ribose) polymerase (PARP) inhibitors have shown efficacy in tumors with homologous recombination repair defects [11]. Furthermore, identifying robust biomarkers indicative of DDR impairment holds great potential for refining patient stratification, allowing for a more personalized approach to DDR-targeted therapies [12]. Combining DDR inhibitors with conventional therapies such as chemotherapy and radiotherapy may enhance therapeutic effectiveness, counteract resistance mechanisms, and ultimately improve clinical outcomes for NSCLC patients [12].

This review provides an in-depth analysis of the upstream and downstream genetic regulators involved in the DDR network of NSCLC. By dissecting the molecular mechanisms driving resistance to radiotherapy and chemotherapy, we aim to identify novel therapeutic targets within the DDR framework. A more comprehensive understanding of these pathways may facilitate the development of integrated treatment approaches capable of mitigating drug resistance and enhancing long-term survival in NSCLC patients.

## 2. DDR Signaling Pathway

**Figure 1:**
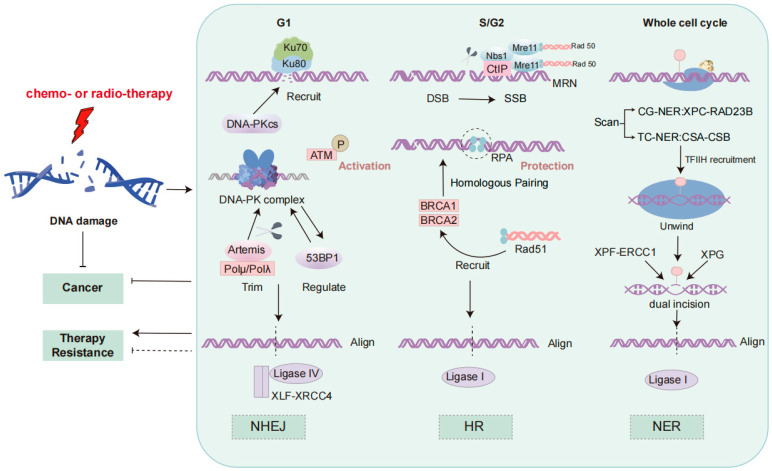
DNA damage response signaling pathways. The figure depicts the three DNA repair mechanisms: NHEJ, HR, and NER. NHEJ, active in G1, is initiated by Ku70/Ku80 binding, followed by DNA-PKcs recruitment, ATM activation, and DNA end processing by Artemis and DNA polymerases. Ligation is mediated by the XRCC4-XLF complex and DNA ligase IV. HR, predominant in S/G2, involves end resection by the MRN complex and CtIP, ssDNA stabilization by RPA, and Rad51-mediated strand invasion facilitated by BRCA1/BRCA2. DNA ligase I completes repair, ensuring genomic integrity. NER includes global genomic NER (XPC-RAD23B) and transcription-coupled NER (CSA-CSB). TFIIH unwinds DNA, XPG and ERCC1-XPF excise the lesion, and the gap is filled by DNA polymerases and sealed by DNA ligase I. ATM, Ataxia–Telangiectasia Mutated; BRCA, Breast Cancer; CtIP, C-terminal Binding Protein-Interacting Protein; DDR, DNA Damage Response; DNA-PKcs, DNA-Dependent Protein Kinase Catalytic Subunit; DSB, Double-Strand Break; HR, Homologous Recombination; Ku70/Ku80, Ku Autoantigen 70/80 kDa; MRN, Mre11-Rad50-Nbs1 Complex; NHEJ, Non-Homologous End Joining; Pol, DNA Polymerase; RPA, Replication Protein A; ssDNA, Single-Stranded DNA; XLF, XRCC4-Like Factor; NER, Nucleotide Excision Repair; TFIIH, Transcription Factor IIH; ERCC1, Excision Repair Cross-Complementation Group 1.

The DNA damage response is a critical mechanism by which cells detect and repair DNA damage, thereby maintaining genomic stability [13]. Three major DNA repair pathways, including homologous recombination (HR), non-homologous end joining (NHEJ), and nucleotide excision repair (NER), play distinct roles in counteracting various forms of DNA damage, and their dysfunction has been implicated in resistance to therapy in NSCLC [14]. These processes are shown in Figure 1.

NHEJ, which functions rapidly and is particularly active during the G1 phase, does not require a homologous template, often leading to insertions or deletions (indels) [15]. The repair process begins with the recognition and binding of broken DNA ends by the Ku70/Ku80 heterodimer [16]. This is followed by the recruitment of the DNA-dependent protein kinase catalytic subunit (DNA-PKcs), forming the DNA-PK complex with Ku, which stabilizes the DNA termini [16]. Ataxia–telangiectasia mutated (ATM) kinase is activated through phosphorylation, initiating the DNA damage response and activating downstream repair factors [16]. Artemis, in cooperation with DNA polymerases μ/λ (Polμ/Polλ), processes the non-complementary ends by trimming the DNA ends to resolve mismatched termini [17]. Finally, the XRCC4-like factor (XLF)-XRCC4 scaffold proteins form a complex with DNA ligase IV, mediating DNA end alignment through microhomology sequences to complete ligation [17]. This process is regulated by 53BP1, which prevents excessive end resection [15]. Due to its error-prone nature, excessive reliance on NHEJ in NSCLC can lead to mutations and genomic instability, contributing to therapeutic resistance, especially to radiotherapy [18].

In contrast, HR is a precise repair mechanism that depends on a homologous template, predominantly active during the S and G2 phases [15]. HR utilizes sister chromatids or homologous chromosomes as templates to repair the broken region, thereby avoiding the mutations that may result from NHEJ [15]. During HR, the MRN complex (Mre11-Rad50-Nbs1) and CtIP first process the DNA ends, generating single-stranded DNA (ssDNA) [19]. The ssDNA is then coated by Replication Protein A (RPA), which protects it from degradation [20]. ATM kinase is activated and recruits BRCA1 and BRCA2, which cooperate to promote the recruitment of Rad51 [20]. Rad51 forms nucleoprotein filaments on the ssDNA, facilitating strand invasion and exchange between the broken DNA ends and the homologous template [20]. This homologous strand exchange precisely restores the broken DNA sequence. Finally, DNA ligase I completes the ligation of the repaired DNA, finalizing the repair process [20]. Deficiencies in HR, such as BRCA1/2 mutations or altered Rad51 expression, are known contributors to genomic instability and are associated with both increased sensitivity to PARP inhibitors and resistance to certain chemotherapeutic agents in NSCLC [18].

Nucleotide excision repair (NER) is another vital pathway, responsible for removing bulky, helix-distorting lesions, such as those induced by ultraviolet (UV) radiation and platinum-based drugs like cisplatin [21]. NER operates via two subpathways: global genomic NER (GG-NER), which scans the entire genome, and transcription-coupled NER (TC-NER), which specifically targets lesions in actively transcribed genes [21]. Lesion recognition is mediated by the XPC-RAD23B complex in GG-NER or CSA-CSB in TC-NER, followed by local DNA unwinding by the transcription factor IIH (TFIIH) complex [21,22]. Dual incisions flanking the lesion are made by the XPG and ERCC1-XPF endonucleases, after which the gap is filled by DNA polymerases and sealed by DNA ligase I [21]. In NSCLC, overexpression of excision repair cross-complementation group 1 (ERCC1) has been strongly associated with resistance to cisplatin, making NER a critical contributor to chemoresistance and a potential therapeutic target [22].

## 3. Radiotherapy

The DDR plays a crucial role in conferring resistance to radiotherapy in NSCLC. Radiotherapy exerts its cytotoxic effects primarily by generating reactive oxygen species (ROS), which induce DSBs, ultimately leading to genomic instability and cell death [23]. However, NSCLC cells have evolved efficient mechanisms to counteract this damage by activating DDR pathways, predominantly relying on NHEJ and HR, to restore genomic integrity and mitigate radiation-induced cytotoxicity [23]. Beyond its direct involvement in DNA repair, DDR also safeguards NSCLC cells by modulating cell cycle checkpoints, thereby preventing the premature mitotic entry of damaged cells [24]. A critical regulatory axis in this process involves checkpoint kinases CHK1 and CHK2, which phosphorylate and inhibit cell division cycle 25C (CDC25C), thereby suppressing cyclin-dependent kinase (CDK) activity [25]. This cascade induces G2/M phase arrest, allowing additional time for DNA repair and ultimately promoting cell survival under radiation-induced stress [25]. The tumor microenvironment (TME) further modulates DDR capacity, creating a supportive niche for NSCLC cells to withstand radiotherapy. Hypoxia, a hallmark of solid tumors, stabilizes hypoxia-inducible factor-1α (HIF-1α), which in turn upregulates key DDR components, strengthening cellular defenses against radiation-induced genotoxic stress [26]. Additionally, nuclear factor kappa-B (NF-κB) signaling, activated within the TME, further augments DDR processes, facilitating cell survival and resistance to apoptosis under genotoxic pressure [26,27].

**Figure 2:**
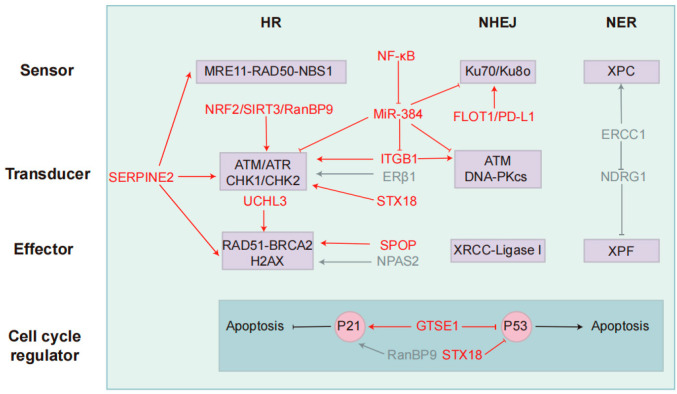
Key targets and modulators in the DDR pathway that regulate resistance to radio- and chemotherapy. NSCLC primarily utilizes three DNA repair pathways: Homologous recombination, non-homologous end joining, and nucleotide excision repair. Each pathway is categorized into three functional components: sensors (damage recognition), transducers (signal amplification/kinase activation), and effectors (repair execution). P53 and P21 interact to regulate cell cycle progression and apoptosis. Key regulatory factors and their functions are mapped to their respective components. Red indicates radiotherapy-associated regulators, while blue represents chemotherapy-associated regulators. These factors target core DDR components, influencing NSCLC resistance to radiotherapy and chemotherapy.

Given the pivotal role of DDR in NSCLC radio-resistance, targeting DDR pathways has emerged as a compelling strategy to enhance radiosensitivity. Pharmacological inhibitors of key DDR regulators, including PARP, ATM/Ataxia telangiectasia and Rad3-related protein (ATR), and CHK1/2, have demonstrated the potential to impair DNA repair processes, disrupt radio-resistance mechanisms, and sensitize NSCLC cells to radiation therapy [6,16,28]. Moreover, recent research has expanded beyond canonical DDR regulators, exploring upstream signaling molecules that modulate DDR activation and downstream effectors that influence cellular responses to DNA damage. To comprehensively understand the molecular determinants of NSCLC radio-resistance and identify novel therapeutic targets, we summarize the upstream and downstream DDR-associated signaling molecules implicated in modulating radiotherapy resistance and potential therapeutic strategies (Table 1) (Figure 2).

### 3.1. FLOT1-PD-L1

Programmed death ligand-1 (PD-L1) is a transmembrane protein that plays a pivotal role in immune regulation by inhibiting T cell-mediated responses through its interaction with PD-1, a key component of the immune checkpoint pathway [29]. In cancer, the overexpression of PD-L1 is often linked to suppressing T cell-mediated immune activity [29]. However, recent research by Shu et al. identified PD-L1 (CD274) as a key gene contributing to lung cancer cell resistance to ionizing radiation (IR) through a genome-wide CRISPR/Cas9 screen [30]. This is consistent with the previously reported results that IR-induced DSB upregulates PD-L1 in cancer cells [31]. The study revealed that IR-induced PD-L1 translocates to the nucleus after deglycosylation via transporter CMTM6, enhancing its interaction with Ku’s core domain, thus participating in the NHEJ repair mechanism [30,31]. This interaction promotes DSB repair, contributing to enhanced radio-resistance, particularly during the G1 and S phases of the cell cycle when NHEJ activity is predominant [30]. Further investigations have identified Flotillin-1 (FLOT1) as a positive regulator of PD-L1, modulating its expression at the cellular level [32]. Notably, FLOT1 depletion leads to a significant reduction in PD-L1 levels, attenuates IR-induced epithelial–mesenchymal transition (EMT), and impairs cancer cell migration, ultimately increasing radiation-induced DNA damage [32]. Further studies showed that FLOT1 depletion activated pro-inflammatory signaling pathways, enhancing the production of C-C motif chemokine ligand 5 (CCL5) and C-X-C motif chemokine 10 (CXCL10), molecules that drive the chemotaxis of CD8^+^ T lymphocytes [32]. This reprograms the tumor immune microenvironment and triggers anti-tumor immune responses [32]. Correlations between FLOT1 expression and immune cell infiltration in NSCLC further underscore its significance in immune regulation [32]. Thus, FLOT1 significantly influences the tumor’s response to radiation therapy by regulating PD-L1 expression and impacting DDR.

Mouse studies have demonstrated that anti-PD-L1 therapy enhances the efficacy of IR via cytotoxic T cell-dependent mechanisms [33,34]. For instance, combining anti-PD-L1 treatment with radiotherapy effectively overcame radio-resistance in a breast cancer model [34]. Additionally, Wang et al. suggested that inhibiting FLOT1 in combination with STING agonists, which promote both innate and adaptive immunity, could potentially rescue the suboptimal effects of STING agonist monotherapy [32,35]. Thus, co-targeting FLOT1 and PD-L1 may offer novel biomarkers and therapeutic strategies for mitigating radiation resistance in NSCLC, ultimately improving radiotherapy outcomes. However, further preclinical and clinical studies are needed to validate the efficacy and safety of such combination strategies, as well as to identify patient populations most likely to benefit.

### 3.2. UCHL3

Ubiquitin C-terminal hydrolase L3 (UCHL3) is a deubiquitinase involved in diverse cellular processes, including cell cycle regulation, signaling, DNA repair, and stress responses [36]. Studies indicate that UCHL3 modulates key DDR factors, such as Ku, Rad51, and BRCA2, primarily through ubiquitination [37,38]. Additionally, UCHL3 stabilizes the aryl hydrocarbon receptor (AHR) via deubiquitination, leading to increased PD-L1 expression and enhanced immune evasion in tumor cells [39]. Liu et al. reported that UCHL3 is upregulated in NSCLC tissues and correlates with poor patient prognosis [40]. UCHL3 promotes the formation of Rad51 foci through deubiquitination and further facilitates interactions between Rad51 and BRCA2, contributing to the DDR [40]. Upon IR exposure, UCHL3 expression increases, and its silence significantly enhances NSCLC radiosensitivity by increasing IR-induced DNA damage and reducing HR repair efficiency [40]. Specifically, UCHL3 knockdown increases IR-induced DNA damage and reduces HR repair efficiency [40].

Furthermore, Xu et al. discovered that LINC00665, via the miR-582-5p/UCHL3/AHR axis, reduces the radiosensitivity of NSCLC cells, thereby aiding tumor immune evasion and resistance to radiotherapy [39]. Silencing LINC00665 or overexpressing miR-582-5p sensitizes NSCLC cells to IR and suppresses tumor immune escape both in vitro and in vivo. The UCHL3 inhibitor perifosine effectively targets triple-negative breast cancer by disrupting the Rad51-BRCA2 axis in both in vitro and in vivo models [41]. In particular, the combination of perifosine and the PARP inhibitor Olaparib has shown better efficacy, emphasizing the importance of inhibiting the DDR pathway in cancer treatment [41]. In summary, UCHL3 maintains the radiation resistance of NSCLC cells by promoting Rad51-mediated HR repair. Targeting UCHL3 may provide a novel strategy to enhance radiotherapy efficacy and improve clinical outcomes in NSCLC. However, given the complexity of DDR regulation and tumor immune microenvironments, further studies are necessary to evaluate the therapeutic window, off-target effects, and optimal patient selection criteria before UCHL3-targeted strategies can be translated into clinical application.

### 3.3. SERPINE2

Serpin family E member 2 (SERPINE2) is a protein belonging to the serine proteinase inhibitor (Serpin) family [42]. In tumors, the upregulation of SERPINE2 expression is typically associated with tumor invasiveness, metastatic potential, and radio-resistance [43,44,45]. Studies have shown that SERPINE2 is upregulated in radio-resistant NSCLC cells, and its knockdown reduces radiation-induced G2/M arrest while inhibiting cell migration and invasion [46,47]. Moreover, SERPINE2 regulates DSB repair through interaction with ATM and MRE11 via non-competitive binding, particularly within the HR repair pathway, by activating Rad51 to promote DNA repair [46]. Mouse models further indicate that high SERPINE2 expression correlates with poor prognosis in lung adenocarcinoma (LUAD) patients [46]. Moreover, various studies have reported that SERPINE2 functions as a regulator that responds to IR and contributes to radio-resistance [48,49]. Given its prognostic significance in LUAD, SERPINE2 is a promising therapeutic target for overcoming radio-resistance. Collecting patient serum SERPINE2 levels may provide valuable prognostic and predictive insights for radiotherapy efficacy in LUAD [50]. However, no large-scale data collection has yet to be performed, warranting further investigation.

### 3.4. NF-κB-miR-384-ITGB1

MicroRNA (miRNA) is a class of small non-coding RNAs, typically 20–24 nucleotides in length, which regulate gene expression by binding to the 3′ untranslated region (3′ UTR) of target mRNAs [51]. This binding leads to either inhibition of translation or degradation of the mRNA [51]. Recent studies have highlighted the critical involvement of miRNAs in tumor progression and resistance to therapies [52]. For instance, miR-384 is significantly downregulated in both NSCLC cell lines and tumor samples [53]. Sun et al. indicated that miR-384 enhances radiosensitivity in NSCLC cells by inhibiting DSB repair, impairing G2/M cell cycle arrest, and promoting cell death [53]. It exerts this effect by directly targeting ATM, Ku70, and Ku80, thereby suppressing both HR and NHEJ repair pathways, leading to increased cell death following radiation therapy [53]. Moreover, radiation therapy downregulates miR-384 expression through the NF-κB pathway, indicating that NF-κB may serve as an upstream regulator of miR-384 [53]. NF-κB suppresses miR-384 expression via binding to the promoter of miR-384, contributing to radio-resistance in NSCLC [53].

Integrin β1 (ITGB1) is an important integrin family member, belonging to the β subunit family [54]. It combines with different α subunits to form various types of integrin complexes and mainly interacts with upregulated ECM proteins in tumors [54]. It is overexpressed in various cancers and links to drug resistance in cancer cells, such as lung, breast, and pancreatic cancer [54,55,56]. Inhibition of ITGB1 enhances radiosensitivity and increases DNA repair damage, especially in HNSCC and pancreatic cancer cells [57,58]. In NSCLC, ITGB1 overexpression correlates with radio-resistance, while its knockdown restores radiosensitivity [59]. ITGB1 affects cell cycle progression, the DDR, and apoptosis, contributing to radio-resistance by promoting EMT via Yes-associated protein 1 (YAP1) activation and regulating the ATM/CHK2 pathway [59]. Additionally, research from the PathCards database shows that ITGB1 is involved in both NHEJ and HR repair pathways and interacts directly or indirectly with multiple key proteins [59]. In conclusion, ITGB1 plays a critical role in NSCLC radio-resistance, and targeting it may offer a novel therapeutic strategy and prognostic biomarker. Recently, ITGB1 was reported to be the target of miR-384 [60]. Studies have shown that Curcumin inhibits the progression of NSCLC by regulating the Circular RNA (circRNA)/miR-384/ITGB1 axis. Specifically, circRNA hsa_circ_0007580 (circ-PRKCA) acts as a miR-384 “sponge,” potentially regulating ITGB1 expression by interacting with various miRNAs, thereby influencing NSCLC development [60]. Given that circRNA/miRNA has been found to enhance radiosensitivity in other cancers, Curcumin may contribute to increasing the radiosensitivity of NSCLC [61]. However, further investigations are needed to fully explore these therapeutic possibilities.

### 3.5. NRF2

Nuclear factor erythroid 2-related factor 2 (NRF2) is a key transcription factor that governs cellular responses to oxidative stress and protects against damage caused by ROS [62]. Elevated NRF2 levels in NSCLC are strongly linked to poor patient prognosis [63]. Research has shown that NRF2 enhances cellular antioxidant capacity through its antioxidant function and independently contributes to the DDR. It facilitates the phosphorylation of replication protein A 32 (RPA32) and recruits DNA topoisomerase II binding protein 1 (TOPBP1) to DSB sites, thereby promoting efficient DNA repair [63]. Additionally, NRF2 activates the ATR/CHK1 signaling pathway, further promoting HR independently of its transcriptional activity [63]. The overexpression of NRF2 confers radio-resistance, leading to enhanced DNA repair capacity and resistance to radiation therapy [63]. IR is a stimulus for NRF2 activation. In breast cancer, the transcription of NRF2 is dose-dependent in response to gamma radiation [64]. Additionally, NRF2-deficient mouse embryonic fibroblasts exhibit increased radiosensitivity [64]. In addition, Chen et al. found that low-dose ionizing radiation enhances radio-resistance in LUAD cells by increasing ROS levels and activating the autophagy/NRF2-HO-1 pathway [65]. Inhibition of autophagy also suppressed radio-resistance and the upregulation of NRF2 and heme oxygenase 1 (HO-1) [65]. The ROS scavenger N-acetyl-L-cysteine (NAC) also prevents the autophagic process induced by low-dose radiation and inhibits NRF2 and HO-1 upregulation, leading to reduced radio-resistance [65]. Therefore, NRF2 plays an essential role in the radiation resistance of NSCLC and represents a promising target for therapeutic strategies aimed at overcoming treatment resistance. However, given its physiological role in protecting normal cells from oxidative stress, systemic inhibition of NRF2 may lead to unintended toxicity. Thus, precise modulation of NRF2 activity, rather than complete inhibition, may be a more viable strategy in overcoming radio-resistance while minimizing side effects.

### 3.6. SPOP

Speckle-type POZ protein (SPOP) is a critical protein involved in preserving genomic integrity and facilitating DNA repair mechanisms [66]. It plays a significant role in regulating DNA repair and cell cycle progression, with implications in various cancers, including prostate and cervical cancer [66]. In LUAD, SPOP is widely expressed, and its levels are elevated in response to DNA damage [67,68]. The knockdown of SPOP by shRNA reduces the cells’ DNA repair capacity, leading to the accumulation of DSBs and subsequently affecting cell cycle checkpoints and apoptosis pathways [67]. SPOP actively regulates the DNA repair process by interacting with DDR factors such as Rad51, particularly in HR repair [67]. Furthermore, studies have shown that SERPINA3 plays a role in inhibiting lung cancer progression through the SPOP/NF-κB signaling axis, suggesting that targeting SPOP could provide a promising therapeutic approach to enhance radiation therapy outcomes in LUAD [69]. Interestingly, recent studies suggest that SPOP also modulates immune responses. Maprotiline, a repurposed antidepressant, was shown to downregulate PD-L1 by targeting SPOP, thereby enhancing antitumor immunity when combined with anti-cytotoxic T-lymphocyte-associated protein 4 (CTLA4) therapy [70]. In conclusion, while targeting SPOP may improve LUAD response to radiotherapy by disrupting HR repair, its role in immune regulation warrants cautious evaluation to avoid undesired effects on tumor immunity or genomic stability.

### 3.7. SIRT3

Sirtuin 3 (SIRT3) is a NAD-dependent deacetylase belonging to the Sirtuin family, which plays an essential role in regulating aging and cellular processes [71]. Other members of the Sirtuin family, such as SIRT1, SIRT6, and SIRT7, have been linked to the DDR and the regulation of radio-resistance in cancer [72,73]. SIRT3 has been reported to exert its effects on lung cancer through various mechanisms. It reduces cisplatin resistance by modulating the Forkhead box O3 (FOXO3)/chromatin licensing and DNA replication factor 1 (CDT1) axis [74]. Additionally, under hypoxic conditions, SIRT3 influences lung cancer progression through the ROS/formyl peptide receptor-1 (FPR1)/HIF-1α axis [75]. Recent studies have highlighted the significant role of SIRT3 in enhancing radiation resistance in lung cancer. The overexpression of SIRT3 confers resistance to radiation therapy, while its silencing sensitizes cells to radiation [76]. Specifically, SIRT3 facilitates the repair of radiation-induced DNA damage and promotes G2/M phase cell cycle arrest, thereby enhancing cellular tolerance to radiation [76]. In SIRT3-deficient cells, the repair of radiation-induced DSBs is delayed, with more γ-H2AX foci, indicative of impaired DNA repair capacity [76]. Moreover, SIRT3 is essential for activating the ATM-CHK2 signaling pathway in HR repair, which allows cells to more effectively repair radiation-induced DNA damage [76]. Despite its role in DNA repair and resistance to radiation therapy, SIRT3 also exhibits both oncogenic and tumor-suppressive properties, suggesting complex dual functions that warrant further investigation to better understand its contributions to chemotherapy and radiotherapy resistance. In addition to DNA repair, SIRT3 also regulates metabolism in NSCLC. Aspirin inhibits tumor growth by activating the AMPK/SIRT3/HK-II pathway, leading to hexokinase 2 (HK-II) dissociation from mitochondria, impaired glycolysis, and mitochondrial dysfunction [77]. This highlights SIRT3’s dual role in radio-resistance and metabolic regulation, offering a potential therapeutic target.

### 3.8. GTSE1

G2 and S phase-expressed 1 (GTSE1) is a protein associated with cell cycle regulation, particularly in the G1/S phase transition [78]. It modulates cell cycle progression by interacting with cyclin-dependent kinase inhibitor 1A (p21), stabilizing p21, and consequently inhibiting the activity of CDK1/2 [79]. Furthermore, GTSE1 has a suppressive effect on the tumor suppressor p53 by facilitating its movement from the nucleus to the cytoplasm in conjunction with mouse double minute 2 homolog (MDM2). This interaction leads to the ubiquitination and degradation of p53, thereby affecting essential pathways related to cell division and apoptosis [80]. These processes influence critical signaling pathways involved in cell cycle regulation and cell death. Recent research has underscored the significant role of GTSE1 in various cancers, including lung, liver, and gastric cancers [81,82,83]. In NSCLC, GTSE1 has been shown to localize to DNA damage sites following radiation exposure, triggering the DNA damage response and facilitating DNA repair [83]. Lei et al. demonstrated that knockdown of GTSE1 expression significantly enhances the radiosensitivity of NSCLC cells, leading to increased DNA damage, inhibited cell proliferation, and enhanced apoptosis [83]. Furthermore, GTSE1 knockdown suppresses the activation of DDR pathways, particularly HR repair, providing new evidence for its role in radiation resistance [83].

In addition to gene silencing, recent studies have identified small-molecule inhibitors targeting GTSE1. Among them, the pyrimidine-2,4-diamine analogue Y18 exhibited strong anticancer effects in colorectal cancer (CRC) HCT116 and NSCLC A549 cells by inducing persistent DNA damage, cell cycle arrest, and senescence [84]. Y18 also inhibited cell adhesion, migration, and invasion in vitro, and suppressed tumor growth in vivo with low toxicity. Mechanistically, Y18 downregulated GTSE1 at both transcriptional and protein levels [84]. These findings support GTSE1 as a promising therapeutic target and Y18 as a potential lead compound for GTSE1-overexpressing cancers, though further studies are needed to assess long-term efficacy and resistance.

### 3.9. STX18

Syntaxin 18 (STX18) is a key protein involved in retrograde vesicular transport between the Golgi apparatus and the endoplasmic reticulum (ER), playing a significant role in cellular processes related to the maintenance of cellular homeostasis and stress response [85]. STX18 influences both cell cycle checkpoints and DNA damage repair mechanisms, thereby potentially reducing the susceptibility of tumor cells to radiation therapy [85]. Research has demonstrated that silencing STX18 expression in A549 and H460 NSCLC cells reduces their resistance to radiation, primarily by disrupting the ATR/CHK1 signaling pathway and affecting the stability of the p53 protein [86,87]. This disruption of normal cell cycle regulation increases the risk of premature mitotic entry [86]. Although the direct effect of STX18 on DNA damage repair mechanisms remains unclear, its role in radio-resistance underscores its potential as a therapeutic target. However, it is important to recognize that STX18 may also impact other biological processes associated with tumor progression, such as EMT and cell migration [86]. Although STX18 shows promise in regulating radiation response, further investigations and clinical studies are needed to confirm its applicability and therapeutic potential in cancer treatment.

### 3.10. RanBP9

Ran Binding Protein 9 (RanBP9) is a recently identified target of ATM kinase and plays a pivotal role in the DDR. Research indicates that, upon IR exposure, RanBP9 accumulates within the nucleus in an ATM kinase-dependent manner, contributing to the activation of DDR pathways [88]. The knockdown of RanBP9 significantly impairs HR repair and enhances radiation-induced cellular senescence, indicating its crucial role in helping cancer cells survive under genotoxic stress and its impact on lung cancer cells’ response to DNA damage and radiation sensitivity [88]. Additionally, RanBP9 has been found to interact with ATM and other associated proteins, such as KAT5/Tip60, to synergistically promote ATM activation, thereby enhancing DNA repair mechanisms [89]. While it is known that KAT5 acetylates ATM to facilitate its activation, the exact molecular mechanism remains unclear [90].

## 4. Chemotherapy

**Figure 3:**
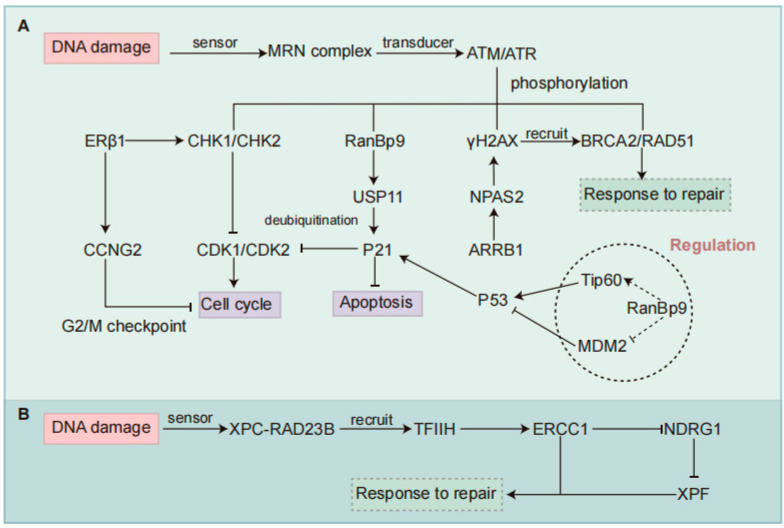
Regulatory mechanisms of DDR pathways involved in chemotherapy resistance in NSCLC. (**A**) Regulation of the HR pathway. ERβ1 enhances chemotherapy sensitivity by promoting G2-M cell cycle arrest via activation of checkpoint kinases CHK1/CHK2 and upregulation of CCNG2. NPAS2 contributes to HR repair and chemoresistance by stabilizing H2AX mRNA, promoting γH2AX accumulation, and increasing phosphorylation of ATM and CHK2. ARRB1 functions as an upstream regulator of NPAS2 to promote its expression. RanBP9 is phosphorylated by ATM under genotoxic stress and translocates to the nucleus, where it stabilizes p21 through USP11-mediated deubiquitination, thereby maintaining cell cycle arrest independently of p53. RanBP9 may also interact with Tip60 or MDM2 to enhance p53 stability and function. (**B**) Regulation of the NER pathway. In the NER pathway, ERCC1 forms a repair complex with XPF to remove damaged DNA. High ERCC1 expression enhances DNA repair capacity and downregulates NDRG1, contributing to chemoresistance. NSCLC, Non-small Cell Lung Cancer; DDR, DNA Damage Response; HR, Homologous Recombination; NER, Nucleotide Excision Repair; ERβ1, Estrogen Receptor β1; CHK, Checkpoint Kinase; CCNG2, Cyclin G2; NPAS2, Neuronal PAS Domain Protein 2; ARRB1, Arrestin Beta 1; ATM, Ataxia Telangiectasia Mutated; RanBP9, Ran-binding Protein 9; USP11, Ubiquitin-specific Peptidase 11; MDM2, Murine Double Minute 2; ERCC1, Excision Repair Cross-complementation Group 1; NDRG1, N-myc Downstream-regulated Gene 1.

The DDR is a fundamental cellular mechanism that plays a pivotal role in chemotherapy resistance by detecting, signaling, and repairing DNA lesions induced by cytotoxic agents. Chemotherapeutic drugs, such as platinum-based compounds (e.g., cisplatin) and topoisomerase inhibitors, exert their cytotoxic effects primarily by inducing DNA crosslinks, SSBs, and DSBs [91]. However, an intact or upregulated DDR facilitates the efficient repair of these lesions, thereby mitigating chemotherapy-induced genomic instability and promoting tumor cell survival [91]. Key DDR pathways, including HR, NHEJ, and NER, contribute to chemotherapy resistance through enhanced DNA repair capacity [92]. Overexpression of HR-associated proteins, such as Rad51 and BRCA1, has been implicated in platinum resistance by promoting the faithful repair of DNA damage [92]. Additionally, DDR activation induces cell cycle arrest via checkpoint kinases (CHK1/2), allowing tumor cells to repair damage before progressing through the cell cycle, further reducing chemotherapy efficacy [92]. Given the pivotal role of DDR in resistance mechanisms, targeting DDR components, such as ATR, DNA-PK, and PARP inhibitors, presents a promising approach to increasing chemotherapy sensitivity and enhancing therapeutic outcomes in NSCLC. To further understand the molecular basis of chemotherapy resistance and uncover new therapeutic targets, the following sections will delve into key DDR-related signaling pathways involved in modulating drug response in NSCLC and potential therapeutic strategies (Table 2) (Figure 2 and Figure 3).

### 4.1. ERCC1/NDRG1

Excision repair cross-complementation group 1 (ERCC1) plays a crucial role in the DDR, particularly within the NER pathway [93]. Together with XPF, ERCC1 forms a repair complex responsible for removing damaged DNA fragments and repairing DSB [93]. The expression levels of ERCC1 are directly correlated with a cell’s ability to repair DNA lesions, making it a significant player in the development of chemotherapy resistance, particularly to drugs such as cisplatin [93]. Studies have shown that silencing ERCC1 in cisplatin/sodium glycididazole (CMNa)-treated NSCLC cells increases DNA damage and apoptosis [94]. ERCC1 downregulates N-Myc downstream-regulated gene 1 (NDRG1), which suppresses the DNA damage response and apoptosis [94]. NDRG1 has been found to act as a tumor suppressor in a variety of tumors [95,96]. Low NDRG1 levels are found in cisplatin-resistant cells [94]. RNA-Seq analysis reveals that in ERCC1-deficient cells, cisplatin/CMNa treatment significantly alters genes involved in apoptosis, DNA repair, and hypoxia [94]. NDRG1 links DNA repair and apoptosis pathways, and ERCC1-mediated downregulation of NDRG1 contributes to cisplatin/CMNa resistance by enabling hypoxia tolerance and preventing apoptosis [94]. Consequently, targeting both ERCC1 and NDRG1 presents a promising therapeutic strategy to combat chemotherapy resistance in lung cancer.

Cisplatin resistance in NSCLC is regulated not only at the transcriptional level but also post-transcriptionally. The spliceosomal factor small nuclear ribonucleoprotein polypeptide A (SNRPA) promotes ERCC1 exon 8 inclusion, facilitating ERCC1-XPF complex formation and DNA repair [97]. Its knockdown induces exon skipping and reverses resistance. m^6^A readers (e.g., IGF2BP1) and RNA-binding proteins (e.g., ELAVL1) stabilize SNRPA mRNA, reinforcing this resistance axis [97]. siRNA targeting ERCC1-E8(+) has shown efficacy in reducing resistance, highlighting the therapeutic relevance of splicing modulation. Beyond splicing, pharmacological strategies also show promise [97]. Demethoxycurcumin (DMC), a bioactive curcuminoid, selectively induces cytotoxicity in NSCLC cells by downregulating ERCC1 via the PI3K/Akt/Snail pathway and suppressing thymidine phosphorylase (TP) expression [98]. It also enhances cisplatin-induced apoptosis by increasing the Bax/Bcl-2 ratio and activating caspase-3, suggesting its dual action on resistance initiation and execution [98]. NDRG1 represents another resistance-modulating factor. While it generally acts as a tumor suppressor and can be upregulated by iron chelators like thiosemicarbazones, its role appears context-dependent, potentially switching to pro-oncogenic behavior via interactions with WNT signaling or phosphatase and tensin homolog (PTEN) [99]. This pleiotropy complicates its therapeutic targeting. In summary, integrating ERCC1-targeted strategies, including splicing modulation and small-molecule inhibition, with context-aware regulation of NDRG1 may offer a multifaceted approach to overcoming cisplatin resistance. However, most findings remain preclinical, and challenges such as splicing plasticity and NDRG1’s dual functions must be addressed before clinical translation.

### 4.2. ERβ1

Estrogen receptor beta 1 (ERβ1), a member of the estrogen receptor family, plays a context-dependent role in cancer progression by regulating proliferation, apoptosis, and gene expression [100,101]. Unlike ERα, which has a more restricted tissue distribution, ERβ1 is widely expressed across different tissues and has been implicated in the progression of various cancers [100]. Though often viewed as a tumor suppressor in breast, prostate, and colon cancer, its role in NSCLC remains controversial [102,103]. In NSCLC, the prognostic value of ERβ1 varies by sex and subcellular localization. High nuclear ERβ1 correlates with better survival in male patients but worse outcomes in females [104,105,106]. Moreover, its cytoplasmic accumulation rather than nuclear localization has been correlated with worse prognosis, highlighting the functional divergence based on receptor compartmentalization [104,105,106]. This variability carries important implications for treatment stratification. For instance, nuclear ERβ1 positivity may identify male patients who could derive greater benefit from EGFR tyrosine kinase inhibitors, while its cytoplasmic predominance may serve as a negative predictor, warranting closer monitoring or alternative therapeutic strategies [107,108].

Specifically, ERβ1 has been shown to promote G2-M phase cell cycle arrest in NSCLC cells, thereby increasing their sensitivity to chemotherapy [109]. ERβ1 upregulates the activity of checkpoint kinases CHK1 and CHK2, prolonging the G2-M phase arrest induced by chemotherapy [109]. Additionally, cyclin G2 (CCNG2), an estrogen-regulated gene, is upregulated in response to ERβ1, controlling the G2-M cell cycle checkpoint [109]. Notably, ERβ1 enhances the cytotoxic effects of chemotherapy agents in p53-deficient NSCLC cells [109,110]. Furthermore, ERβ1 facilitates tumor invasion through a non-genomic mechanism by transcriptionally activating thioredoxin-related transmembrane protein 4 (TMX4), which elevates circ-TMX4 levels [111]. Circ-TMX4 acts as a sponge for miR-622, resulting in upregulation of CXCR4, a key mediator of metastasis, thus establishing the ERβ1/circ-TMX4/miR-622/CXCR4 axis as a novel pathway linking ERβ1 to tumor aggressiveness and therapy response [111].

In addition, pharmacological network analyses have identified ERβ as a potential target of traditional Chinese medicine formulations such as Mahuang FuziXixin Decoction (MFXD), which downregulates ERβ expression and its interactions with oncogenic factors, including epidermal growth factor receptor (EGFR), HIF1α, and RELA [112]. These findings suggest that ERβ1 functions not only as a prognostic marker but also as a therapeutic node with multi-target relevance. Collectively, the evidence supports the integration of ERβ1 profiling, considering both sex and subcellular distribution, into personalized NSCLC treatment algorithms. Nonetheless, its dualistic behavior underscores the necessity for standardized detection protocols and stratified clinical validation to establish ERβ1 as a reliable biomarker or therapeutic target.

### 4.3. NPAS2

Neuronal PAS Domain Protein 2 (NPAS2) is a core circadian clock gene that not only regulates biological rhythms but also plays a significant role in tumor progression [113]. In LUAD, NPAS2 is frequently overexpressed and correlates with poor overall survival [113,114]. Functionally, NPAS2 promotes proliferation, migration, invasion, and EMT of LUAD cells [113]. Notably, it also facilitates metabolic reprogramming by enhancing aerobic glycolysis, a metabolic hallmark of cancer, thereby supporting rapid tumor growth [115]. Silencing NPAS2 suppresses glycolysis and promotes mitochondrial oxidative metabolism, resulting in attenuated malignant phenotypes in A549 cells [115]. Upstream, the transcription factor arrestin beta 1 (ARRB1) has been identified as a positive regulator of NPAS2, binding directly to its promoter and enhancing its transcription [115]. Overexpression of ARRB1 can partially rescue the inhibitory effects of NPAS2 knockdown, suggesting a functional ARRB1–NPAS2 axis that drives LUAD progression [115].

Moreover, NPAS2 is involved in DNA damage repair, particularly the HR pathway [114]. It binds to H2AX mRNA and stabilizes it, facilitating the DNA repair cascade and enabling LUAD cells to resist cisplatin-induced cytotoxicity [114]. Functional experiments have demonstrated that NPAS2 knockdown leads to reduced γH2AX accumulation, impaired HR repair capacity, and decreased phosphorylation of key DNA repair proteins such as ATM and CHK2, while having negligible effects on NER pathways [114]. In this regard, NPAS2 not only serves as a transcriptional and metabolic regulator but also as a key modulator of DNA repair fidelity in LUAD [114]. In addition, He et al. demonstrated that NPAS2 polymorphisms are associated with NSCLC prognosis, underscoring its potential as a prognostic biomarker [116]. Collectively, these findings suggest that NPAS2 promotes LUAD progression through the regulation of metabolism, DNA repair, and oncogenic signaling and may serve as a novel therapeutic target. However, current findings regarding NPAS2 are primarily based on in vitro cell lines and limited in vivo mouse models, which may not fully recapitulate the complexity of human LUAD. Moreover, targeting NPAS2 in the HR pathway may raise concerns about potential off-target effects and genomic instability in normal proliferating cells.

### 4.4. RanBP9

Ran Binding Protein 9 (RanBP9), an evolutionarily conserved scaffolding protein, plays crucial roles in diverse cellular processes, including DDR, cell cycle regulation, and protein homeostasis [117,118,119]. Upon genotoxic stress, activation of the ATM signaling pathway leads to phosphorylation of RanBP9 at multiple serine residues, facilitating its nuclear translocation. Within the nucleus, RanBP9 stabilizes the cyclin-dependent kinase inhibitor p21 via a post-translational mechanism [120]. It tethers p21 to the deubiquitinase USP11, promoting USP11-mediated deubiquitination and preventing proteasomal degradation [120]. Notably, this mechanism functions independently of p53 transcriptional activity, providing a parallel regulatory layer to maintain p21 levels during DDR [120]. This RanBP9-USP11-p21 axis is particularly significant in the context of DNA damage, as it enables the rapid, cell cycle-independent stabilization of p21. In contrast, canonical pathways often rely on transcriptional induction of p21 by p53 or are tightly regulated by E3 ubiquitin ligases such as SCF^Skp2^, CRL4^Cdt2^, and APC/C^Cdc20^, which degrade p21 in a cell cycle-dependent manner [120]. Compared to these mechanisms, the RanBP9-mediated pathway allows cells to bypass transcriptional delays and mount an immediate protective response to DNA insults.

Further insights into RanBP9 biology reveal that its regulatory scope may extend beyond p21. Although RanBP9 does not appear to physically interact with p53, its absence leads to blunted ATM activity and reduced phosphorylation of p53 at Ser15, indirectly impairing p53 stability and function [88,121]. Mechanistically, this may involve crosstalk with key modulators such as Tip60, which acetylates and stabilizes p53, or potentially MDM2, the principal E3 ligase for p53 degradation. Thus, RanBP9 may fine-tune the balance between DDR signaling and apoptosis through a broader network than previously appreciated [88]. Functionally, loss of RanBP9 impairs DNA repair, reduces p21 expression, and increases the sensitivity of NSCLC cells to genotoxic agents such as ionizing radiation and cisplatin [88]. Interestingly, RanBP9-deficient cells also show heightened responsiveness to PARP and ATR inhibitors but not ATM inhibitors, suggesting a synthetic vulnerability reminiscent of a “BRCAness-like” phenotype [122,123]. This opens up therapeutic possibilities: tumors with low RanBP9 expression may benefit from PARP inhibitor-based strategies, while targeting the RanBP9-USP11-p21 interaction could sensitize resistant tumors to DNA-damaging agents.

Nevertheless, these insights must be interpreted with caution. While RanBP9 contributes to therapy resistance by enhancing DNA repair and cell survival, it also safeguards genomic integrity. Targeting RanBP9 may sensitize tumor cells but could also affect normal tissue homeostasis, especially under stress. Therefore, any therapeutic intervention must strike a balance between exploiting tumor vulnerabilities and preserving physiological repair mechanisms, highlighting the need for context-specific modulation rather than blanket inhibition.

## 5. Summary and Prospects

The DNA damage response network plays a paradoxical role in non-small cell lung cancer. While essential for preserving genomic stability, aberrant activation of DDR components contributes to resistance to chemotherapy and radiotherapy. Key mediators such as ERCC1, NDRG1, and NPAS2 have been linked to platinum-based chemotherapy resistance, whereas FLOT1, PD-L1, UCHL3, and NRF2 have been implicated in radio-resistance. Emerging evidence from preclinical models highlights promising therapeutic targets, including RNA splicing factors like SNRPA, circadian regulators such as NPAS2, and pathways bridging immune response and DNA repair through FLOT1-PD-L1 and UCHL3-AHR interactions. However, the clinical translation of these findings remains constrained by several major challenges.

A primary concern lies in balancing therapeutic efficacy with toxicity. Many DDR-related proteins, such as RanBP9 and GTSE1, are also critical for the homeostasis of normal proliferating cells, raising the risk of systemic toxicity. Approaches like transient, pulsed inhibition or tumor-targeted delivery using nanoparticles may help to mitigate these effects. Another obstacle is the complexity of biomarker discovery and application. The prognostic relevance of ERβ1 appears to vary with sex, NPAS2 influences metabolic reprogramming, and PD-L1 has been observed to translocate into the nucleus under certain conditions. These observations emphasize the need for dynamic, spatially resolved biomarkers, potentially utilizing liquid biopsy techniques to monitor splicing variants or artificial intelligence tools to assess subcellular protein localization [124]. In addition, the redundancy and context-dependency of DNA repair pathways often undermine single-agent strategies. Suppression of homologous recombination through inhibition of factors like SPOP or UCHL3 may be offset by compensatory upregulation of non-homologous end joining mechanisms, as observed in the FLOT1-Ku interaction. This necessitates rational combination therapies. For example, tumors with low RanBP9 expression may benefit from co-administration of PARP and ATR inhibitors, while combining STING agonists with FLOT1 inhibition could concurrently target immune evasion and DNA repair plasticity.

Looking ahead, a precision framework for DDR intervention should integrate mechanistic synergy and technological innovation. Synthetic lethality, such as the combination of ERCC1 deficiency with hypoxia-targeted agents and coordinated modulation of the DDR, immune, and metabolic pathways may offer enhanced therapeutic potential. Spatial multi-omics could help delineate DDR-immune niches within tumors, as exemplified by SERPINE2-ATM aggregates at invasive fronts, while CRISPR-based functional screens may uncover alternative vulnerabilities arising after DDR inhibition [125]. Finally, the design of clinical trials must evolve to incorporate enriched biomarker stratification. This could include DDR-defect signatures such as Rad51 foci to guide UCHL3-targeted therapy, sex- or hormone-dependent factors like ERβ1 expression, and metabolic imaging to assess NPAS2-driven glycolytic shifts.

In conclusion, the DDR landscape in NSCLC represents both a therapeutic opportunity and a biological challenge. Capitalizing on its potential requires a context-aware, multi-target approach that embraces both the preservation of genomic integrity and the exploitation of cancer-specific vulnerabilities. Future progress will depend on the development of more predictive models, improved delivery systems, and adaptive clinical strategies that can translate biological insights into meaningful clinical benefit.

## Figures and Tables

**Table 1:** Key targets in NSCLC radio-resistance and potential therapeutic strategies.

Target/Pathway	Function/Mechanism	Radio-Resistance Role	Therapeutic Strategies	Major Challenges	References
FLOT1-PD-L1	FLOT1 regulates PD-L1 expression; PD-L1-Ku interaction enhances DNA repair	Promotes NHEJ repair; suppresses CD8^+^ T-cell infiltration	Anti-PD-L1 antibodies + RT; FLOT1 inhibitors + STING agonists	Immune-related adverse events; standardization of nuclear PD-L1 assessment	[29,30,31,32,33,34,35]
UCHL3	Deubiquitinase stabilizing Rad51/BRCA2; upregulates PD-L1 via AHR	Enhances HR repair; drives immune evasion	UCHL3 inhibitors (e.g., Perifosine) + PARP inhibitors	Off-target effects; cross-regulation with the AHR–PD-L1 axis	[36,37,38,39,40,41]
SERPINE2	Binds ATM/MRE11 to activate Rad51; induces G2/M arrest	Facilitates HR repair; promotes metastasis	SERPINE2 inhibitors; serum SERPINE2 as biomarker	Lack of high-affinity inhibitors; association with invasive phenotypes	[42,43,44,45,46,47,48,49,50]
NF-κB-miR-384-ITGB1	miR-384 targets ATM/Ku70/Ku80; ITGB1 activates YAP1/ATM-CHK2	Suppresses HR/NHEJ; promotes EMT	miR-384 restoration (Curcumin); ITGB1/circRNA targeting	Delivery efficiency of miRNAs; complex role of ITGB1 in the EMT	[51,52,53,54,55,56,57,58,59,60,61]
NRF2	Activates ATR/CHK1 and RPA32/TOPBP1; boosts antioxidant/HR pathways	Enhances ROS scavenging and HR repair	NRF2 inhibitor (e.g., ML385) + radiotherapy; NAC to reverse resistance	Protective role of NRF2 in normal tissue; dose-dependent effects	[62,63,64,65]
SPOP	Regulates HR repair via Rad51 interaction; stabilizes DDR components	Promotes HR repair; suppresses apoptosis	Maprotiline (SPOP modulator) + immunotherapy	Dual effect on NF-κB pathway; genomic instability risk	[66,67,68,69,70]
SIRT3	NAD-dependent deacetylase; activates ATM-CHK2 signaling in HR repair	Enhances HR repair; induces G2/M arrest	Aspirin activates the AMPK/SIRT3/HK-II axis; SIRT3 inhibitors (e.g., AGK7)	Dual role in metabolism and DNA repair; systemic effects of SIRT3 modulation	[71,72,73,74,75,76,77]
GTSE1	Stabilizes p21; suppresses p53 via MDM2-mediated degradation	Facilitates HR repair; inhibits DDR checkpoint activation	Y18 (GTSE1 inhibitor) + DNA-damaging agents	Efficacy differs by p53 status; long-term toxicity unknown	[78,79,80,81,82,83,84]
STX18	Activates ATR/CHK1 signaling; reduces p53 stability	Enhances HR repair; promotes EMT and cell migration	siRNA-mediated STX18 silencing + radiotherapy; ER-Golgi transport inhibition (e.g., Brefeldin A analogs)	Ubiquitin-dependent regulation may disrupt secretory pathways; potential link to EMT	[85,86,87]
RanBP9	ATM-dependent DDR activator; synergizes with KAT5 for ATM activation	Enhances HR repair; delays senescence	RanBP9 inhibition; combined ATM/KAT5 targeting	Genomic stability concerns in normal tissue; lack of specific inhibitors	[88,89,90]

DDR, DNA Damage Response; EMT, Epithelial–Mesenchymal Transition; ER, Endoplasmic Reticulum; GTSE1, G2 and S Phase-Expressed 1; HR, Homologous Recombination; ITGB1, Integrin Beta 1; KAT5, Lysine Acetyltransferase 5 (Tip60); MDM2, Mouse Double Minute 2 Homolog; miRNA, MicroRNA; NAC, N-Acetyl-L-Cysteine; NF-κB, Nuclear Factor Kappa Beta; NHEJ, Non-Homologous End Joining; NSCLC, Non-Small Cell Lung Cancer; PARP, Poly(ADP-Ribose) Polymerase; PD-L1, Programmed Death-Ligand 1; RPA32, Replication Protein A 32; ROS, Reactive Oxygen Species; RT, Radiation Therapy; SERPINE2, Serpin Family E Member 2; SIRT3, Sirtuin 3; STING, Stimulator of Interferon Genes; TOPBP1, DNA Topoisomerase II Binding Protein 1; YAP1, Yes-Associated Protein 1; NRF2, Nuclear Factor Erythroid 2–related Factor 2; SPOP, Speckle-type POZ Protein; FLOT1, Flotillin-1; STX18,Syntaxin 18; RanBP9, RAN Binding Protein 9; UCHL3, Ubiquitin Carboxyl-Terminal Hydrolase L3; ATM, Ataxia Telangiectasia Mutated; ATR, Ataxia Telangiectasia and Rad3-Related; AHR, Aryl Hydrocarbon Receptor; AMPK, AMP-activated Protein Kinase; HK-II, Hexokinase II; NAD, Nicotinamide Adenine Dinucleotide; CHK, Checkpoint Kinase.

**Table 2:** Key targets in NSCLC chemoresistance and potential therapeutic strategies.

Target/Pathway	Function/Mechanism	Resistance Role	Therapeutic Strategies	Major Challenges	References
ERCC1/NDRG1	The ERCC1-XPF complex mediates NER/DSB repair; ERCC1 suppresses NDRG1	Promotes cisplatin resistance via hypoxia tolerance and apoptosis inhibition	siRNA targeting the ERCC1-E8(+) isoform; DMC inhibiting PI3K/Akt/Snail; iron chelators (e.g., thiosemicarbazones) to upregulate NDRG1	Hematologic toxicity; splicing plasticity contributing to resistance; dual role (tumor suppressor/promoter); microenvironment dependency	[93,94,95,96,97,98,99]
ERβ1	Upregulates CHK1/CHK2; induces G2-M arrest via cyclin G2	Enhances chemotherapy sensitivity (cisplatin/doxorubicin) in p53-deficient cells	SERMs; traditional formula MFXD targeting the ERβ/EGFR/HIF1α axis	Gender and subcellular localization affecting efficacy; pro-metastatic risk via circ-TMX4/CXCR4	[100,101,102,103,104,105,106,107,108,109,110,111,112]
NPAS2	Stabilizes H2AX mRNA to activate HR repair; regulates circadian rhythm	Drives cisplatin resistance via HR repair and glycolysis	CRISPR knockout or small-molecule inhibitors; combination with PARPi leveraging the “BRCAness” phenotype	Circadian disruption from systemic inhibition; glycolytic reprogramming may impair efficacy	[113,114,115,116]
RanBP9	Scaffolds the ATM-p21-USP11 axis; stabilizes p21 via deubiquitination	Supports the DDR and chemoresistance by enhancing DNA repair	Disrupting RanBP9-USP11 interaction; combination with ATR or PARP inhibitors	Genomic stability concerns in normal tissue; lack of specific inhibitors	[117,118,119,120,121,122,123]

HR, Homologous Recombination; DDR, DNA Damage Response; ATM, Ataxia Telangiectasia Mutated; ERCC1, Excision Repair Cross-Complementation Group 1; NDRG1, N-Myc Downstream-Regulated Gene 1; NER, Nucleotide Excision Repair; NPAS2, Neuronal PAS Domain Protein 2; USP11, Ubiquitin-Specific Peptidase 11; DMC, Demethoxycurcumin; SERMs, Selective Estrogen Receptor Modulators; ATR, Ataxia Telangiectasia and Rad3-related Protein; PARP, Poly (ADP-ribose) Polymerase; MFXD, Mahuang FuziXixin Decoction; RanBP9, Ran Binding Protein 9; ERβ1, Estrogen Receptor beta 1; CHK, Checkpoint Kinases; CXCR4, C-X-C Chemokine Receptor Type 4; EGFR, Epidermal Growth Factor Receptor; TMX4, Thioredoxin-Related Transmembrane Protein 4.
